# *N*′-(2-Hydroxy­benzyl­idene)-2-methoxy­benzohydrazide monohydrate

**DOI:** 10.1107/S1600536808024483

**Published:** 2008-08-06

**Authors:** Jiu-Fu Lu, Suo-Tian Min, Xiao-Hui Ji, Zhong-Hai Dang

**Affiliations:** a School of Chemistry and Environmental Science, Shaanxi University of Technology, Hanzhong 723000, People’s Republic of China

## Abstract

In the title compound, C_15_H_14_N_2_O_3_·H_2_O, the Schiff base mol­ecule is approximately planar, with a dihedral angle between the two aromatic rings of 10.2 (3)°. The mol­ecular structure is stabilized by O—H⋯N and N—H⋯O hydrogen bonds. In the crystal structure, the Schiff base and water mol­ecules are linked together by inter­molecular O—H⋯O hydrogen bonds, forming chains parallel to the *a* axis.

## Related literature

For general background on Schiff bases derived from condensation of aldehydes with benzohydrazides, see: Fun *et al.* (2008 ▶); Alhadi *et al.* (2008 ▶); Ali *et al.* (2007 ▶); Zou *et al.* (2004 ▶); Shan *et al.* (2008 ▶); Bedia *et al.* (2006 ▶); Terzioglu & Gürsoy (2003 ▶). For related structures, see: Nie (2008 ▶); He (2008 ▶); Shi *et al.* (2007 ▶). For bond-length data, see: Allen *et al.* (1987 ▶).
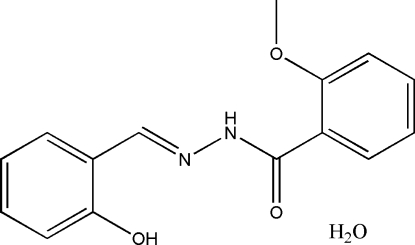


## Experimental

### 

#### Crystal data

C_15_H_14_N_2_O_3_·H_2_O
*M* *_r_* = 288.30
Orthorhombic, 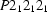

*a* = 4.761 (2) Å
*b* = 14.035 (3) Å
*c* = 21.073 (4) Å
*V* = 1408.1 (7) Å^3^
*Z* = 4
Mo *K*α radiation
μ = 0.10 mm^−1^
*T* = 298 (2) K
0.17 × 0.16 × 0.15 mm

#### Data collection

Bruker APEXII CCD area-detector diffractometer
Absorption correction: multi-scan (*SADABS*; Sheldrick, 2004 ▶) *T*_min_ = 0.983, *T*_max_ = 0.985
11662 measured reflections
1808 independent reflections
1345 reflections with *I* > 2σ(*I*)
*R*_int_ = 0.047

#### Refinement

*R*[*F* ^2^ > 2σ(*F* ^2^)] = 0.049
*wR*(*F* ^2^) = 0.130
*S* = 1.06
1808 reflections
201 parameters
4 restraints
H atoms treated by a mixture of independent and constrained refinement
Δρ_max_ = 0.19 e Å^−3^
Δρ_min_ = −0.24 e Å^−3^

### 

Data collection: *APEX2* (Bruker, 2004 ▶); cell refinement: *SAINT* (Bruker, 2004 ▶); data reduction: *SAINT*; program(s) used to solve structure: *SHELXS97* (Sheldrick, 2008 ▶); program(s) used to refine structure: *SHELXL97* (Sheldrick, 2008 ▶); molecular graphics: *SHELXTL* (Sheldrick, 2008 ▶); software used to prepare material for publication: *SHELXTL*.

## Supplementary Material

Crystal structure: contains datablocks global, I. DOI: 10.1107/S1600536808024483/ci2646sup1.cif

Structure factors: contains datablocks I. DOI: 10.1107/S1600536808024483/ci2646Isup2.hkl

Additional supplementary materials:  crystallographic information; 3D view; checkCIF report

## Figures and Tables

**Table 1:** Hydrogen-bond geometry (Å, °)

*D*—H⋯*A*	*D*—H	H⋯*A*	*D*⋯*A*	*D*—H⋯*A*
O1—H1⋯N1	0.82	1.97	2.669 (3)	143
N2—H2⋯O3	0.90 (1)	1.97 (3)	2.629 (3)	129 (3)
O4—H4*B*⋯O2	0.88 (3)	2.01 (3)	2.880 (4)	171 (3)
O4—H4*A*⋯O2^i^	0.88 (3)	2.04 (2)	2.893 (4)	165 (4)

Symmetry code: (i) 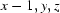.

**Fig. 1.**
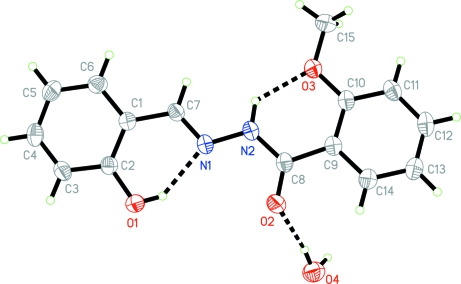
The molecular structure of the title compound, showing 30% probability displacement ellipsoids and the atom-numbering scheme.

**Fig. 2.**
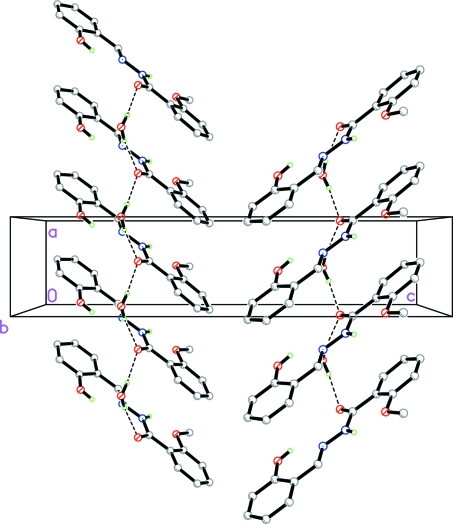
The crystal packing of the title compound, viewed approximately along the b axis.

## supplementary crystallographic information

### Comment

Schiff bases derived from the condensation of aldehydes with benzohydrazides have been widely investigated, either for their structures (Fun *et al.*, 2008; Alhadi *et al.*,  2008; Ali *et al.*,  2007; Zou *et al.*,  2004; Shan *et al.*,  2008) or for their biological properties (Bedia *et al.*,  2006; Terzioglu & Gürsoy, 2003). This study extends the structural study on such compounds. We report here the crystal structure of the title new Schiff base compound.

The asymmetric unit of the title compound consists of a Schiff base molecule and a water molecule of crystallization (Fig. 1). The bond lengths are within normal values (Allen *et al.*,  1987), and are comparable to the values observed in similar compounds (Nie, 2008; He, 2008; Shi *et al.*, 2007). The dihedral angle between the two aromatic rings in the Schiff base molecule is 10.2 (3)°, indicating that the molecule is approximately coplanar. The molecular structure is stabilized by O—H···N and N—H···O hydrogen bonds.

In the crystal structure (Fig. 2), the Schiff base and water molecules are linked into chains running parallel to the *a* axis by intermolecular O—H···O hydrogen bonds (Table 1).

### Experimental

The title compound was prepared by the Schiff base condensation of salicylaldehyde (0.1 mol) and 2-methoxybenzohydrazide (0.1 mmol) in ethanol (50 ml). The excess ethanol was removed by distillation. The colourless solid obtained was filtered and washed with ethanol. Single crystals suitable for X-ray diffraction were grown by slow evaporation from an ethanol solution at room temperature.

### Refinement

The imino and water H atoms were located in a difference map and refined with N–H, O–H, and H···H distances restrained to 0.90 (1), 0.85 (1), and 1.37 (2) Å, respectively. The other H atoms were positioned geometrically [C–H = 0.93-0.96 Å and O–H = 0.82 Å] and refined using a riding model, with U_iso_(H) = 1.2U_eq_(C) and 1.5U_eq_(C15 and O1). In the absence of significant anomalous scattering, Friedel pairs were merged.

### Crystal data

**Table:**

C_15_H_14_N_2_O_3_·H_2_O	*F*_000_ = 608
*M**_r_* = 288.30	*D*_x_ = 1.360 Mg m^−^^3^
Orthorhombic, *P*2_1_2_1_2_1_	Mo *K*α radiation λ = 0.71073 Å
Hall symbol: P 2ac 2ab	Cell parameters from 1886 reflections
*a* = 4.761 (2) Å	θ = 2.5–24.3º
*b* = 14.035 (3) Å	µ = 0.10 mm^−^^1^
*c* = 21.073 (4) Å	*T* = 298 (2) K
*V* = 1408.1 (7) Å^3^	Block, colourless
*Z* = 4	0.17 × 0.16 × 0.15 mm

### Data collection

**Table:**

Bruker APEXII CCD area-detector diffractometer	1808 independent reflections
Radiation source: fine-focus sealed tube	1345 reflections with *I* > 2σ(*I*)
Monochromator: graphite	*R*_int_ = 0.047
*T* = 298(2) K	θ_max_ = 27.0º
ω scans	θ_min_ = 1.7º
Absorption correction: multi-scan(SADABS; Sheldrick, 2004)	*h* = −6→6
*T*_min_ = 0.983, *T*_max_ = 0.985	*k* = −17→17
11662 measured reflections	*l* = −26→26

### Refinement

**Table:**

Refinement on *F*^2^	Secondary atom site location: difference Fourier map
Least-squares matrix: full	Hydrogen site location: inferred from neighbouring sites
*R*[*F*^2^ > 2σ(*F*^2^)] = 0.049	H atoms treated by a mixture of independent and constrained refinement
*wR*(*F*^2^) = 0.130	*w* = 1/[σ^2^(*F*_o_^2^) + (0.0693*P*)^2^ + 0.0505*P*] where *P* = (*F*_o_^2^ + 2*F*_c_^2^)/3
*S* = 1.06	(Δ/σ)_max_ = 0.001
1808 reflections	Δρ_max_ = 0.19 e Å^−^^3^
201 parameters	Δρ_min_ = −0.24 e Å^−^^3^
4 restraints	Extinction correction: none
Primary atom site location: structure-invariant direct methods	

### Special details

**Table:**

Geometry. All esds (except the esd in the dihedral angle between two l.s. planes) are estimated using the full covariance matrix. The cell esds are taken into account individually in the estimation of esds in distances, angles and torsion angles; correlations between esds in cell parameters are only used when they are defined by crystal symmetry. An approximate (isotropic) treatment of cell esds is used for estimating esds involving l.s. planes.
Refinement. Refinement of F^2^ against ALL reflections. The weighted R-factor wR and goodness of fit S are based on F^2^, conventional R-factors R are based on F, with F set to zero for negative F^2^. The threshold expression of F^2^ > 2sigma(F^2^) is used only for calculating R-factors(gt) etc. and is not relevant to the choice of reflections for refinement. R-factors based on F^2^ are statistically about twice as large as those based on F, and R- factors based on ALL data will be even larger.

### Fractional atomic coordinates and isotropic or equivalent isotropic displacement parameters (Å^2^)

**Table:**

	*x*	*y*	*z*	*U*_iso_*/*U*_eq_	
O1	1.0565 (6)	0.25999 (15)	0.11044 (10)	0.0662 (7)	
H1	0.9443	0.2516	0.1394	0.099*	
O2	0.5284 (6)	0.30810 (15)	0.26095 (10)	0.0632 (7)	
O3	0.4681 (5)	0.04698 (14)	0.35246 (10)	0.0600 (6)	
O4	0.0300 (7)	0.40911 (16)	0.22254 (13)	0.0761 (8)	
N1	0.8661 (5)	0.17231 (17)	0.21429 (11)	0.0447 (6)	
N2	0.6938 (6)	0.15858 (18)	0.26624 (11)	0.0475 (6)	
C1	1.2024 (6)	0.1040 (2)	0.14441 (13)	0.0430 (7)	
C2	1.2117 (7)	0.1808 (2)	0.10233 (13)	0.0454 (7)	
C3	1.3847 (8)	0.1756 (2)	0.04953 (14)	0.0596 (10)	
H3	1.3908	0.2266	0.0214	0.072*	
C4	1.5436 (8)	0.0984 (2)	0.03827 (15)	0.0614 (9)	
H4	1.6573	0.0968	0.0024	0.074*	
C5	1.5411 (8)	0.0215 (2)	0.07896 (15)	0.0617 (9)	
H5	1.6533	−0.0314	0.0711	0.074*	
C6	1.3700 (8)	0.0247 (2)	0.13124 (15)	0.0539 (8)	
H6	1.3654	−0.0272	0.1586	0.065*	
C7	1.0217 (7)	0.10236 (19)	0.19971 (13)	0.0450 (7)	
H7	1.0197	0.0484	0.2253	0.054*	
C8	0.5265 (7)	0.2299 (2)	0.28622 (13)	0.0457 (7)	
C9	0.3359 (7)	0.2092 (2)	0.34123 (13)	0.0429 (7)	
C10	0.3100 (7)	0.1214 (2)	0.37297 (13)	0.0470 (7)	
C11	0.1233 (7)	0.1132 (3)	0.42336 (14)	0.0573 (9)	
H11	0.1055	0.0553	0.4445	0.069*	
C12	−0.0358 (8)	0.1904 (3)	0.44229 (15)	0.0637 (9)	
H12	−0.1616	0.1838	0.4758	0.076*	
C13	−0.0103 (8)	0.2763 (2)	0.41233 (14)	0.0565 (8)	
H13	−0.1165	0.3283	0.4255	0.068*	
C14	0.1751 (7)	0.2848 (2)	0.36233 (14)	0.0509 (8)	
H14	0.1925	0.3435	0.3422	0.061*	
C15	0.4542 (10)	−0.0415 (2)	0.38602 (17)	0.0750 (12)	
H15A	0.4918	−0.0306	0.4302	0.112*	
H15B	0.5913	−0.0847	0.3691	0.112*	
H15C	0.2700	−0.0684	0.3813	0.112*	
H2	0.695 (9)	0.0996 (11)	0.2825 (15)	0.080*	
H4A	−0.113 (5)	0.371 (2)	0.2284 (19)	0.080*	
H4B	0.169 (5)	0.373 (2)	0.2359 (18)	0.080*	

### Atomic displacement parameters (Å^2^)

**Table:**

	*U*^11^	*U*^22^	*U*^33^	*U*^12^	*U*^13^	*U*^23^
O1	0.0823 (19)	0.0551 (13)	0.0613 (14)	0.0192 (14)	0.0190 (13)	0.0106 (10)
O2	0.0598 (15)	0.0610 (13)	0.0688 (14)	0.0056 (14)	0.0168 (14)	0.0188 (11)
O3	0.0623 (15)	0.0557 (12)	0.0621 (13)	0.0040 (12)	0.0182 (13)	0.0086 (10)
O4	0.0771 (18)	0.0626 (15)	0.0885 (18)	0.0020 (16)	0.0056 (18)	0.0012 (13)
N1	0.0417 (14)	0.0533 (15)	0.0390 (13)	−0.0045 (13)	0.0031 (12)	−0.0012 (11)
N2	0.0474 (15)	0.0540 (15)	0.0412 (13)	−0.0037 (14)	0.0074 (13)	0.0013 (11)
C1	0.0423 (17)	0.0448 (15)	0.0418 (15)	−0.0052 (14)	−0.0015 (14)	−0.0042 (13)
C2	0.0482 (18)	0.0461 (16)	0.0417 (15)	−0.0004 (16)	0.0002 (14)	−0.0049 (13)
C3	0.069 (2)	0.059 (2)	0.0506 (19)	−0.002 (2)	0.0155 (17)	0.0066 (15)
C4	0.060 (2)	0.075 (2)	0.0495 (18)	0.000 (2)	0.0131 (17)	−0.0117 (17)
C5	0.063 (2)	0.0592 (19)	0.063 (2)	0.0121 (19)	0.0074 (19)	−0.0126 (17)
C6	0.065 (2)	0.0448 (16)	0.0517 (17)	0.0021 (17)	0.0014 (17)	−0.0021 (14)
C7	0.0477 (18)	0.0438 (15)	0.0434 (15)	−0.0057 (16)	0.0056 (15)	0.0007 (12)
C8	0.0374 (17)	0.0563 (17)	0.0433 (15)	−0.0044 (17)	0.0000 (15)	−0.0030 (14)
C9	0.0359 (16)	0.0546 (17)	0.0381 (14)	−0.0061 (14)	−0.0052 (14)	−0.0049 (13)
C10	0.0400 (17)	0.0591 (18)	0.0420 (15)	−0.0043 (16)	0.0003 (14)	−0.0050 (14)
C11	0.053 (2)	0.070 (2)	0.0495 (18)	−0.0070 (19)	0.0073 (16)	0.0069 (16)
C12	0.054 (2)	0.087 (2)	0.0494 (18)	−0.005 (2)	0.0116 (18)	−0.0080 (17)
C13	0.048 (2)	0.071 (2)	0.0506 (17)	0.0055 (19)	0.0003 (18)	−0.0131 (16)
C14	0.0474 (18)	0.0572 (18)	0.0482 (17)	−0.0020 (17)	−0.0032 (17)	−0.0058 (15)
C15	0.085 (3)	0.064 (2)	0.075 (2)	0.012 (2)	0.012 (2)	0.0219 (18)

### Geometric parameters (Å, °)

**Table:**

O1—C2	1.346 (4)	C5—C6	1.371 (4)
O1—H1	0.8200	C5—H5	0.93
O2—C8	1.220 (3)	C6—H6	0.93
O3—C10	1.358 (4)	C7—H7	0.93
O3—C15	1.430 (4)	C8—C9	1.501 (4)
O4—H4A	0.88 (3)	C9—C14	1.383 (4)
O4—H4B	0.88 (3)	C9—C10	1.407 (4)
N1—C7	1.268 (3)	C10—C11	1.389 (4)
N1—N2	1.381 (3)	C11—C12	1.380 (5)
N2—C8	1.347 (4)	C11—H11	0.93
N2—H2	0.897 (10)	C12—C13	1.366 (5)
C1—C6	1.397 (4)	C12—H12	0.93
C1—C2	1.397 (4)	C13—C14	1.380 (4)
C1—C7	1.449 (4)	C13—H13	0.93
C2—C3	1.386 (4)	C14—H14	0.93
C3—C4	1.343 (5)	C15—H15A	0.96
C3—H3	0.93	C15—H15B	0.96
C4—C5	1.378 (4)	C15—H15C	0.96
C4—H4	0.93		
			
C2—O1—H1	109.5	O2—C8—N2	121.9 (3)
C10—O3—C15	119.0 (3)	O2—C8—C9	121.1 (3)
H4A—O4—H4B	101.2 (19)	N2—C8—C9	117.0 (3)
C7—N1—N2	115.5 (2)	C14—C9—C10	118.1 (3)
C8—N2—N1	119.7 (2)	C14—C9—C8	115.7 (3)
C8—N2—H2	125 (3)	C10—C9—C8	126.2 (3)
N1—N2—H2	115 (3)	O3—C10—C11	122.3 (3)
C6—C1—C2	118.1 (3)	O3—C10—C9	118.2 (3)
C6—C1—C7	119.1 (3)	C11—C10—C9	119.5 (3)
C2—C1—C7	122.8 (3)	C12—C11—C10	120.5 (3)
O1—C2—C3	118.1 (3)	C12—C11—H11	119.8
O1—C2—C1	122.7 (3)	C10—C11—H11	119.8
C3—C2—C1	119.2 (3)	C13—C12—C11	120.7 (3)
C4—C3—C2	121.3 (3)	C13—C12—H12	119.7
C4—C3—H3	119.4	C11—C12—H12	119.7
C2—C3—H3	119.4	C12—C13—C14	119.1 (3)
C3—C4—C5	121.1 (3)	C12—C13—H13	120.4
C3—C4—H4	119.4	C14—C13—H13	120.4
C5—C4—H4	119.4	C13—C14—C9	122.2 (3)
C6—C5—C4	118.7 (3)	C13—C14—H14	118.9
C6—C5—H5	120.7	C9—C14—H14	118.9
C4—C5—H5	120.7	O3—C15—H15A	109.5
C5—C6—C1	121.7 (3)	O3—C15—H15B	109.5
C5—C6—H6	119.2	H15A—C15—H15B	109.5
C1—C6—H6	119.2	O3—C15—H15C	109.5
N1—C7—C1	122.0 (3)	H15A—C15—H15C	109.5
N1—C7—H7	119.0	H15B—C15—H15C	109.5
C1—C7—H7	119.0		

### Hydrogen-bond geometry (Å, °)

**Table:**

*D*—H···*A*	*D*—H	H···*A*	*D*···*A*	*D*—H···*A*
O1—H1···N1	0.82	1.97	2.669 (3)	143
N2—H2···O3	0.90 (1)	1.97 (3)	2.629 (3)	129 (3)
O4—H4B···O2	0.88 (3)	2.01 (3)	2.880 (4)	171 (3)
O4—H4A···O2^i^	0.88 (3)	2.04 (2)	2.893 (4)	165 (4)

Symmetry codes: (i) *x*−1, *y*, *z*.
